# A novel mutation of adenomatous polyposis coli (APC) gene results in the formation of supernumerary teeth

**DOI:** 10.1111/jcmm.13303

**Published:** 2017-08-07

**Authors:** Fang Yu, Wenping Cai, Beizhan Jiang, Laijun Xu, Shangfeng Liu, Shouliang Zhao

**Affiliations:** ^1^ Department of Pediatric Dentistry School & Hospital of Stomatology Tongji University Shanghai Engineering Research Center of Tooth Restoration and Regeneration Shanghai China; ^2^ Center for Translational Neurodegeneration and Regenerative Therapy Shanghai Tenth People's Hospital Tongji University School of Medicine Shanghai China; ^3^ Department of Stomatology Huashan Hospital Fudan University Shanghai China; ^4^ Department of Endodontics School & Hospital of Stomatology Shanghai Engineering Research Center of Tooth Restoration and Regeneration Tongji University Shanghai China

**Keywords:** supernumerary teeth, gardner syndrome, adenomatous polyposis coli (APC), mutation

## Abstract

Supernumerary teeth are teeth that are present in addition to normal teeth. Although several hypotheses and some molecular signalling pathways explain the formation of supernumerary teeth, but their exact disease pathogenesis is unknown. To study the molecular mechanisms of supernumerary tooth‐related syndrome (Gardner syndrome), a deeper understanding of the aetiology of supernumerary teeth and the associated syndrome is needed, with the goal of inhibiting disease inheritance *via* prenatal diagnosis. We recruited a Chinese family with Gardner syndrome. Haematoxylin and eosin staining of supernumerary teeth and colonic polyp lesion biopsies revealed that these patients exhibited significant pathological characteristics. APC gene mutations were detected by PCR and direct sequencing. We revealed the pathological pathway involved in human supernumerary tooth development and the mouse tooth germ development expression profile by RNA sequencing (RNA‐seq). Sequencing analysis revealed that an APC gene mutation in exon 15, namely 4292‐4293‐Del GA, caused Gardner syndrome in this family. This mutation not only initiated the various manifestations typical of Gardner syndrome but also resulted in odontoma and supernumerary teeth in this case. Furthermore, RNA‐seq analysis of human supernumerary teeth suggests that the APC gene is the key gene involved in the development of supernumerary teeth in humans. The mouse tooth germ development expression profile shows that the APC gene plays an important role in tooth germ development. We identified a new mutation in the APC gene that results in supernumerary teeth in association with Gardner syndrome. This information may shed light on the molecular pathogenesis of supernumerary teeth. Gene‐based diagnosis and gene therapy for supernumerary teeth may become available in the future, and our study provides a high‐resolution reference for treating other syndromes associated with supernumerary teeth.

## Introduction

Supernumerary teeth are an odontostomatologic anomaly characterized by the existence of an excessive number of teeth in relation to the normal dental formula. The condition could be characterized as single or multiple and as unilateral or bilateral. The condition can occur in the upper or lower jaw 1. Significant variations in tooth eruption site, direction and shape features are noted depending on the location, including increases in the number of middle supernumerary teeth, premolar teeth, molars and teeth distal to molars, as well as teeth conical in shape, nodular teeth, supplementary teeth (similar to normal tooth morphology) and dental tumours 2. Supernumerary teeth can occur in the primary dentition and permanent dentition. The incidence of permanent dentition is 0.1–3.6% 3, 4, 5, and the incidence of primary dentition is 0.3–0.8% 6. Supernumerary teeth are more common in men, with a male: female ratio of approximately 1.3–6.5:1. Supernumerary teeth are more common in maxillary teeth, with an incidence of 75–91% 2. In the normal population, a single extra tooth is the most common manifestation, accounting for 76–86% of cases. More than two extra teeth are found in 12–23% of cases, whereas three or more teeth are noted in only 1% of cases 7.

Hypotheses regarding supernumerary teeth focus on atavism 8, tooth bud dichotomy theory 9, genetic factors 10, 11, 12, 13 and the dental plate overactive doctrine 14.

Signalling pathways related to the regulation of tooth development include Shh, Wnt, FGF, BMP and TNF. In addition, numerous mouse mutations are associated with supernumerary teeth 13.

Many syndromes are associated with supernumerary teeth (Table 1), including Gardner syndrome 14, cleidocranial dysplasia 15, trichorhinophalangeal syndrome 16, 17, oro‐facio‐digital syndrome type I 18, Nance‐Horan syndrome 19, Rothmund–Thomson syndrome 20, Ellis–Van Creveld syndrome 21, craniosynostosis 22, Ehlers‐Danlos syndrome Type III 23 and Robinow syndrome 24.

**Table 1:** Syndromes related to supernumerary teeth

Syndrome	OMIM	Gene
Gardner syndrome	175100	Adenomatous polyposis coli (APC)
Cleidocranial dysplasia	119600	Runt‐related transcription factor 2 (RUNX2)
Trichorhinophalangeal	190350	Trichorhinophalangeal syndrome 1 (TRPS1)
Oro‐facio‐digital type I	311200	Oro‐facio‐digital syndrome 1(OFD1)
Nance‐Horan	302350	Nance Horan syndrome (NHS)
Rothmund–Thomson	268400	RecQ protein‐like 4 (RECQL4)
Ellis–Van Creveld	225500	Ellis–Van Creveld (EVC); EVC2
Craniosynostosis	614188	IL11RA
Ehlers‐Danlos Type III	130020	Collagen type III (COL3A1); Tenascin‐XB
Robinow	180700	ROR2

OMIM, online mendelian inheritance in man.

Gardner syndrome, which is also known as familial colorectal polyposis, is an autosomal dominant form of polyposis characterized by the presence of multiple intracolonic polyps and extracolonic tumours. The extracolonic tumours may include osteomas of the skull, thyroid cancer, epidermoid cysts and fibromas 25, as well as the occurrence of desmoid tumours in approximately 15% of affected individuals. The condition is inherited in an autosomal dominant manner and caused by mutations in the APC gene located on chromosome 5q21 (Online Mendelian Inheritance in Man (OMIM): 175100). Gardner syndrome can be identified based on oral findings, including multiple impacted and supernumerary teeth; multiple jaw osteomas, which give a ‘cotton‐wool’ appearance to the jaws, multiple odontomas, congenital hypertrophy of the retinal pigment epithelium (CHRPE) and multiple adenomatous polyps of the colon. Gardner syndrome is also associated with FAP (Familial Adenomatous Polyposis) and may manifest as aggressive fibromatosis (desmoid tumours) of the retroperitoneum 26.

Although several theoretical hypotheses and various molecular signalling pathways have been implicated in the formation of supernumerary teeth to date, the exact mechanism is unclear.

In our study, we performed clinical and genetic analyses to explore the mutation implicated in a family with Gardner syndrome associated with supernumerary teeth and odontoma. We detected a novel mutation in the APC gene that results in the formation of supernumerary teeth and tumours. This study deepens our understanding of the mechanism and aetiology of supernumerary teeth. Inheritance of this syndrome can potentially be inhibited *via* prenatal diagnosis.

## Materials and methods

### Ethics approval

Blood samples were obtained from patients who were informed of the purpose of the research, and the study was approved by School of Stomatology, Tongji University's ethics committee.

### Case report

A 15‐year‐old male patient (III‐1) from Shanghai visited the department of pedodontics of the Affiliated Stomatology Hospital of Tongji University with a chief complaint of pain in the right posterior mandibular region. The patient's medical and family histories were inconclusive. Patients from this family have had a family history of Gardner syndrome.

### Clinical data

We collected family clinical data using dental panoramic radiographs, computed tomography of teeth, cephalometric radiographs and enteroscopic examination.

### Mutation analysis

After informed consent was obtained per approval by the Medical Ethics Committee of Tongji University School of Stomatology, venous blood samples were obtained from the affected patients and a number of unaffected members of the Gardner syndrome family. An unaffected member [the third uncle (II‐2)] was chosen as the control.

Genomic DNA was extracted from peripheral blood lymphocytes of the family members (QIAGEN DNA Blood Mini Kit; QIAGEN Biotech Ltd, Germany, Duesseldorf). Based on the nucleotide sequence of the adenomatous polyposis coli (APC) gene (GenBank: M74088.1) (http://www.ncbi.nlm.nih.gov/nuccore/M74088.1), we designed 30 pairs of primers for 15 exons (Table 2). The APC gene was amplified by polymerase chain reaction (PCR) and sequenced using our APC primers. ClustalW2 software was used to align the sequencing results (http://www.ebi.ac.uk/Tools/msa/clustalw2), and the DNA sequence was viewed with Chromas software.

**Table 2:** Adenomatous polyposis coli (APC) primers

Exon position	Primer	Forward (5′–3′)	Reverse (5′–3′)	Size(bp)
Exon 1	APC‐1	TAGCATATTAACACAATTCT	CTGAATGAATTCAATATATC	456
Exon 2	APC‐2	TACAGAATCATGTCTTGAAG	GCTGTACTTGGATCTACACA	226
Exon 3	APC‐3	AGAGGAAGTCTAAGGAAGTA	GGAGTACACAAGGCAATGTT	477
Exon 4	APC‐4	GTATTGCTCTTCTGCAGTCT	GTTGTACTGCCAAGTTACTT	198
Exon 5	APC‐5	CATGCACCATGACTGACGTA	CTTAGAAACAAGTAACTTAC	256
Exon 6	APC‐6	TGCGGTGAGCTGAGATTATG	TCTCAGAATAACTACCTATT	401
Exon 7	APC‐7	TGTACTGATGTTAACTCCAT	AGAACCATCTTGCTTCATAC	204
Exon 8	APC‐8	CTTAACATGATGTTATCTGT	AGTCATGGCATTAGTGACCA	209
Exon 9	APC‐9	CCATTCATCACTTAATTGGT	GATGTACACTATAGAGAACA	479
Exon 10	APC‐10	CTCCTAGACTTATTCTAAGA	CACCAGTAATTGTCTATGTC	395
Exon 11	APC‐11	ACCAACTTGGTACCAGTTTG	TAACTCATACCTGAGCTATC	255
Exon 12	APC‐12	TGAGTGAAGTATCAGTTATG	CAGTGAGCTGAGATTGCACA	311
Exon 13	APC‐13	GTGATAGGATTACAGGCGTG	TGAAATTCATATTATAGTAC	300
Exon 14	APC‐14	CATAGAAGTTAATGAGAGAC	CATTGCTTACAATTAGGTCT	353
	APC‐15‐1	GGAGATGTGGAATACTTGGA	TTCTTGAGCATGCTAACTGC	302
	APC‐15‐2	GTGGAATCTCTCAGCAAGAA	GGTAACACTGTAGTATTCAA	444
	APC‐15‐3	GCATCTCATCGTAGTAAGCA	GCTGACACTTCTTCCATGAC	341
	APC‐15‐4	TTGAATACTACAGTGTTACC	AACCATCACTACTACTGACA	442
	APC‐15‐5	GTCATGGAAGAAGTGTCAGC	CCAGAGTTCAACTGCTCATC	449
	APC‐15‐6	TGTCAGTAGTAGTGATGGTT	CATAGTCATCTTCTTGACAC	509
	APC‐15‐7	GATGAGCAGTTGAACTCTGG	ACTTCTACTCTGTGCAGAAC	624
	APC‐15‐8	GTGTCAAGAAGATGACTATG	TCACAGGATCTTCAGCTGAC	575
	APC‐15‐9	GTTCTGCACAGAGTAGAAGT	CTGACAGAAGTACATCTGCT	472
Exon 15	APC‐15‐10	GTCAGCTGAAGATCCTGTGA	CTGAACGGAGCTGGCAATCG	277
	APC‐15‐11	AGCAGATGTACTTCTGTCAG	TCATCATCATCTGAATCATC	560
	APC‐15‐12	CGATTGCCAGCTCCGTTCAG	CCTTGCCACAGGTGGAGGTA	623
	APC‐15‐13	GATGATTCAGATGATGATGA	CTTCTCCAGCAGCTAACTCA	328
	APC‐15‐14	TACCTCCACCTGTGGCAAGG	GTCATCCAATTCAGGTATGG	344
	APC‐15‐15	TGAGTTAGCTGCTGGAGAAG	TACACGTGTCCTATATTCAG	367
	APC‐15‐16	CCATACCTGAATTGGATGAC	AGGTCAACATCATCATCATC	496
	APC‐15‐17	CTGAATATAGGACACGTGTA	GGTATGTCTTTGGATGACTG	475
	APC‐15‐18	GATGATGATGATGTTGACCT	GCATAGCCTGATGCTTGAGG	422
	APC‐15‐19	CAGTCATCCAAAGACATACC	TCCTGAATAGCTTTCCAATC	515
	APC‐15‐20	CCTCAAGCATCAGGCTATGC	CTTGATCAGGTGTAAGATGA	460
	APC‐15‐21	GATTGGAAAGCTATTCAGGA	GACTTGTACTTGAGGAGCTA	436
	APC‐15‐22	TCATCTTACACCTGATCAAG	AGGAGACTGTATAGGTCTAC	529
	APC‐15‐23	TAGCTCCTCAAGTACAAGTC	ACTTCTTGGAATACTACTGG	469
	APC‐15‐24	GTAGACCTATACAGTCTCCT	CTTTCAGAATGAGACCGTGC	652
	APC‐15‐25	CCAGTAGTATTCCAAGAAGT	TCCAGTTCTTCTCCAAGTGC	542
	APC‐15‐26	GCACGGTCTCATTCTGAAAG	ACCATTTGTAGCACCTGAGG	327
	APC‐15‐27	GCACTTGGAGAAGAACTGGA	TTCATTAGTCTCTGATACAG	575
	APC‐15‐28	CCTCAGGTGCTACAAATGGT	ACTGGATTCTGTGCTGTCAG	587
	APC‐15‐29	CTGTATCAGAGACTAATGAA	ACTGTACAAACATACTTGGC	531
	APC‐15‐30	CTGACAGCACAGAATCCAGT	GCTATCTCTATGCACATCAT	675

### RNA‐Seq

To construct the cDNA library, total cellular RNA from human supernumerary teeth [Normal 1: proband's mother; Normal 2: proband's third uncle (II‐2); Patient 1: proband (III‐1); Patient 2: proband's second uncle (II‐1)] dental stem cells and ICR mouse dental germ cells in the embryonic (E) and postnatal (PN) phases (E13.5, E14.5, E16.5, E18.5, PN1, PN3, PN5, PN7) were extracted using Trizol reagent (Invitrogen, USA, California, Carlsbad). Library construction was performed following the manufacturer's suggestions. The libraries were sequenced on the Illumina HiSeq 2000 platform. We used the omicsbean software (http://www.omicsbean.com:88) to focus our human supernumerary teeth bioinformatics analysis on heatmap analysis, gene ontology (GO) analysis and Kyoto Encyclopedia of Genes and Genomes (KEGG). RNA‐seq analysis was performed to identify and visualize the expression of the APC gene in human supernumerary teeth and tooth germ development. We analysed expression levels of gene (CTNNB1, AXIN1, GSK3B, CSNK1E, ARHGEF4) from different supernumerary teeth patients.

## Results

### Family case report

**Figure 1:**
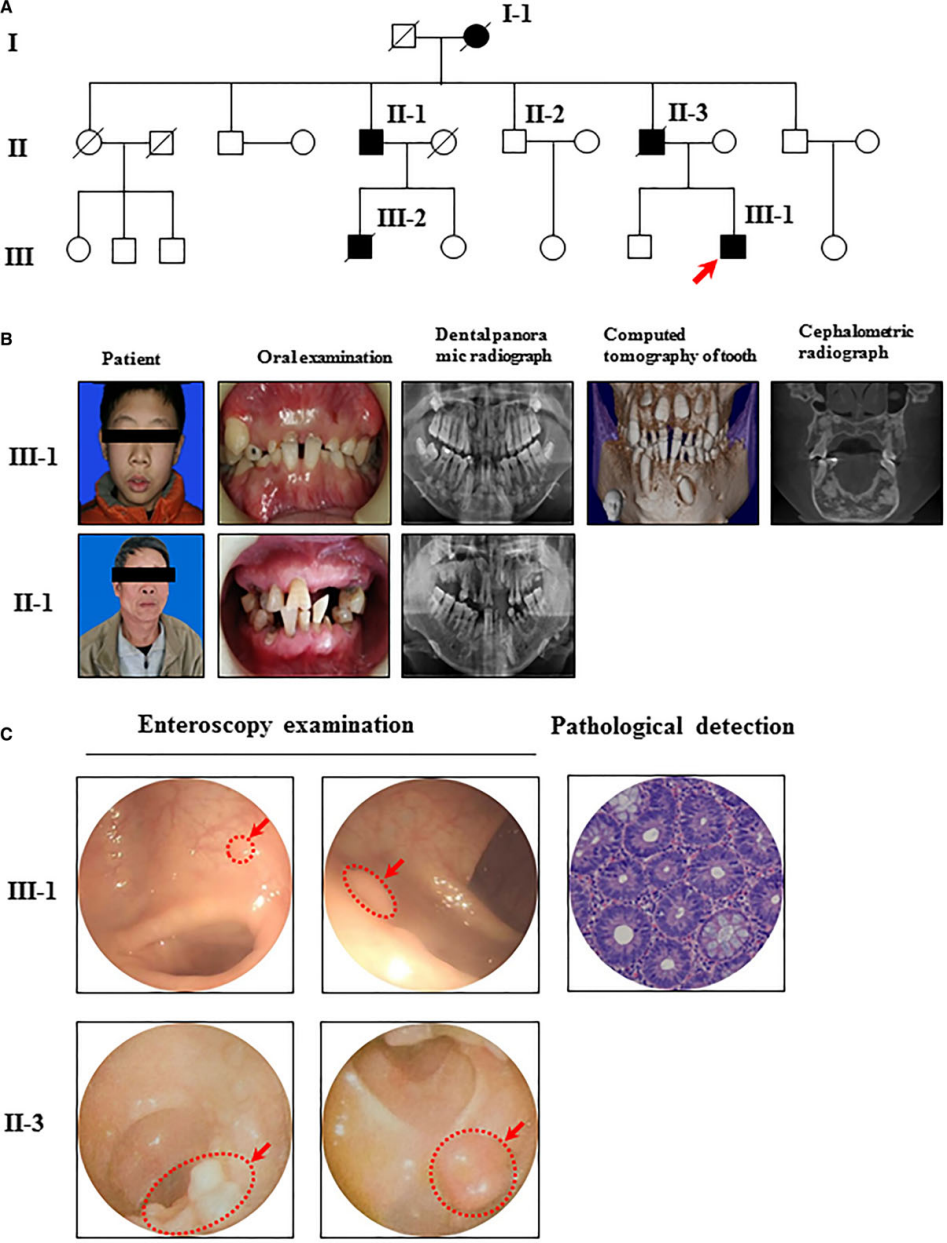
Pedigree of the Gardner syndrome family. (**A**) Pedigree of the proband's family from Shanghai, China. Arrow indicates proband III‐1, a 15‐year‐old male; (**B**) Oral examination of the Gardner syndrome family; (**C**) Enteroscopy examination of the Gardner syndrome family.

In this study of a Gardner syndrome family from Shanghai, the proband (III‐1) was a 15‐year‐old boy. Based on clinical examination and data collection, we drew a pedigree including three generations with a total of 23 people, including five patients with Gardner syndrome [proband (III‐1), proband's father (II‐3), proband's second uncle (II‐1), proband's brother (III‐2) and proband's grandmother (I‐1)]. The morbidity rate of this family is 21.74% (Fig. 1A). The patient's paternal grandmother, father and brother had died of the disease. The second uncle (II‐1) (who is sick) is alive and was diagnosed with FAP. A whole mouth pantomography revealed multiple high‐density clumps in the jaw with teeth and a plurality of permanent impaction tumours.

### Family clinical findings

The proband (III‐1) is a 15‐year‐old boy. Oral examination revealed unerupted teeth (11, 12, 21, 22, 32, 33, 42 and 43), deciduous teeth and dentition (Fig. 1B). A full mouth curved with body plies within the upper and lower jaw was noted. Examination revealed multiple high‐density clumps (Fig. 1B). Preliminary diagnosis was multiple tooth tumours. Dental clinic examination also revealed unerupted teeth (11, 12, 21, 22, 32, 33, 42 and 43), Meng resistance and deciduous teeth (1C, 1B, 1A, 2A, 2B, 2C, 3B, 3C, 4B and 4C). The upper and lower jaws were subject to a cone‐beam computed tomography examination (CBCT) scan. The scan revealed multiple high‐density masses within the upper and lower jaws of different sizes and shapes. Some images revealed normal tissue coated with a clear shadow. Some images revealed no clear boundaries, and some images described teeth shape (Fig. 1B). Combining the results of full mouth curved body plies and CBCT, multiple mandibular osteomas, dental tumours and multiple teeth (with a plurality of permanent Meng resistance and multiple deciduous teeth) were detected. Gardner syndrome was suspected in this patient.

Given the characteristics of familial Gardner syndrome, colonoscopy was recommended. Based on the colonoscopy, we observed ileocecal valve lip‐like presentation with normal systolic and diastolic function. Each section of the colon and rectal mucosa was smooth and organized. The vascular texture was clear with no obvious ulcers, polyps or tumours. The patient was diagnosed with multiple colorectal polyps (Fig. 1C). The colon biopsy report revealed tubular adenomas. Given the clinical manifestations in the proband, the examination and the medical history, the proband was diagnosed with Gardner syndrome.

The proband's father (II‐3) died due to the Gardner syndrome. He was hospitalized and subjected to surgery on several occasions. He had a discharge diagnosis of Gardner syndrome. The pathological diagnosis was moderately differentiated tubular adenocarcinoma with mucinous adenocarcinoma (Fig. 1C).

The proband's second uncle (II‐1) was 66 years old. He was invited to Tongji University Dental Hospital for assessment *via* a routine mouth examination (Fig. 1B). Full mouth curved body pile examination revealed Meng resistance in teeth 13, 21, 22 and 33, as well as multiple high‐density masses in the upper and lower jaws (Fig. 1B). In November 2001, there were complaints of increased stool frequency, and the amount of blood and pus associated with the colonoscopy and biopsy was consistent with rectal adenocarcinoma grade II, FAP and cancer. Colorectal cancer abdominal perineal resection (Miles operation) was performed thrice (March 2002, January 2003 and November 2001), and good recovery was noted.

The proband's third uncle (II‐2) was 63 years old without any clinical symptoms. He had a normal colonoscopy and was invited to Tongji University Dental Hospital for assessment. A full mouth curved layer film revealed normal tooth loss and a lack of jaw osteomas, teeth tumours or other abnormal conditions. Subsequent molecular biology tests revealed no APC gene mutations.

### Mutation analysis

**Figure 2:**
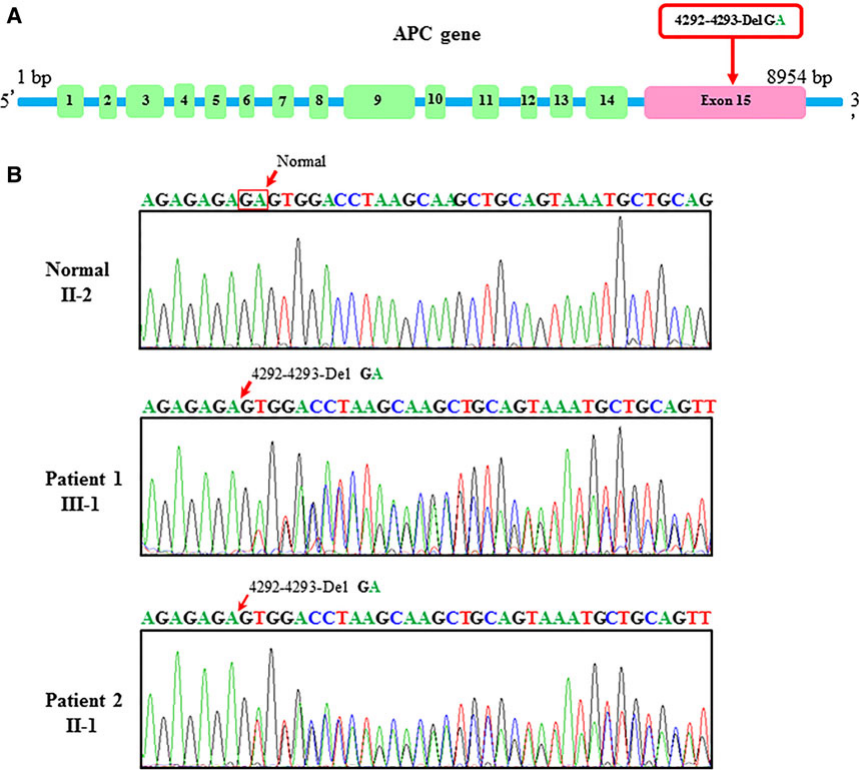
Adenomatous polyposis coli (APC) gene structure and partial DNA sequences of exon 15 of the APC gene from the proband's family. (**A**) APC gene structure; (**B**) The arrow indicates the position of the APC heterozygous mutation 4292‐4293‐Del GA associated with supernumerary teeth in Gardner syndrome patients.

Mutation analysis revealed that APC (Fig. 2A) was the key mutation associated with supernumerary teeth in this family. The mutation was confirmed by PCR and subsequent sequencing of the PCR products, which revealed one mutation in the APC gene when compared with the Single Nucleotide Polymorphism (SNP) Database in NCBI (http://www.ncbi.nlm.nih.gov/snp/) and the Human Gene Mutation Database (http://www.hgmd.org/). The APC gene mutation we reported in this manuscript in exon 15, 4292‐4293‐Del GA caused Gardner syndrome in this family is a novel mutation (Fig. 2B). It will add into Human Gene Mutation Database. This heterozygous mutation not only initiated the various manifestations of typical Gardner syndrome but also resulted in odontoma and supernumerary teeth in this case.

### RNA‐seq analysis

**Figure 3:**
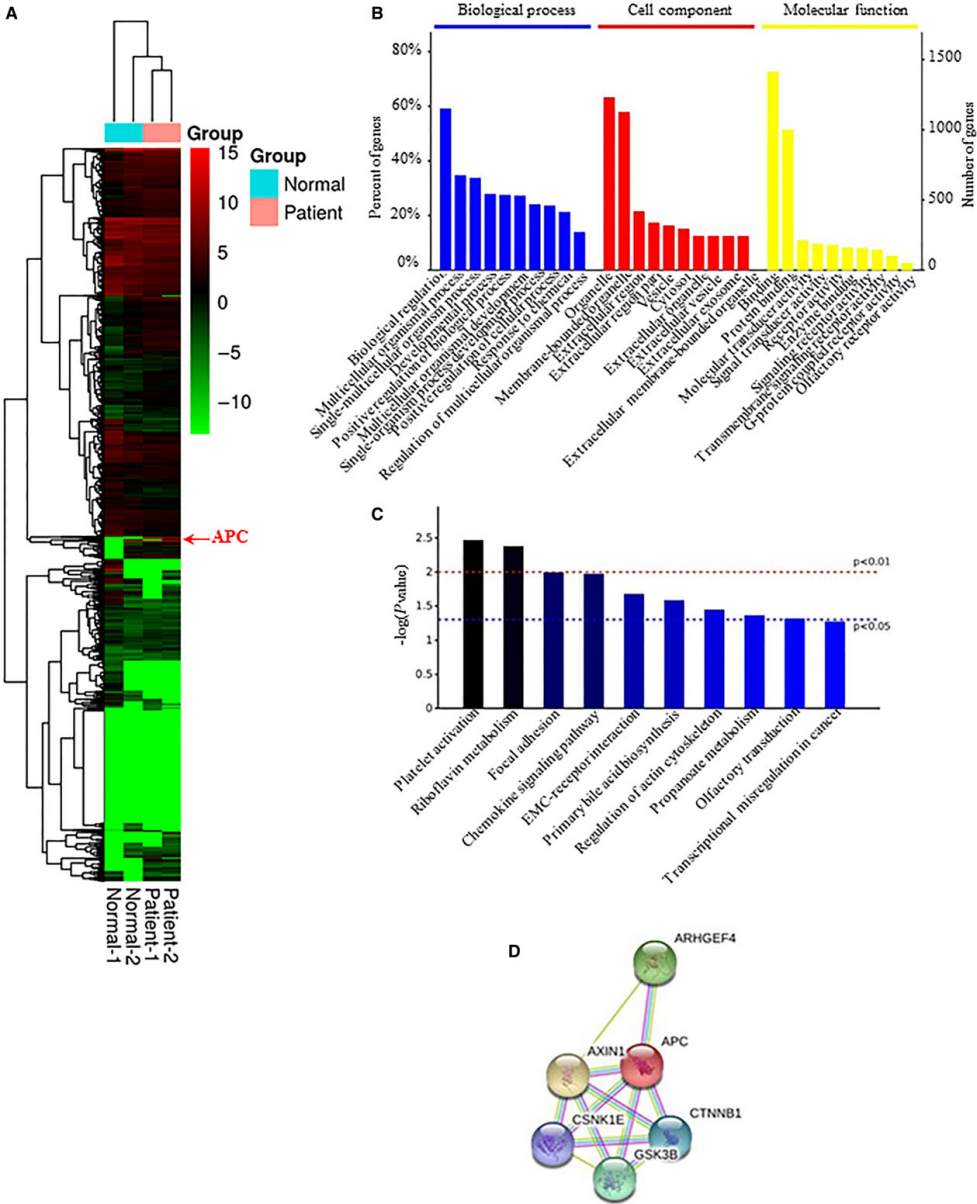
RNA‐seq analysis. (**A**) Heatmap showing the relative expression of activated genes in the supernumerary teeth patients; (**B**) GO analysis; (**C**) KEGG analysis; (**D**) Interacting proteins for APC gene.

The RNA‐seq bioinformatics analysis indicates that the relative expression of activated genes in the supernumerary teeth patients from heatmap (Fig. 3A); GO analysis shows that the important process of supernumerary teeth was biological regulation, multicellular organismal progress, organelle, membrane‐bounded organelle, binding and protein binding (Fig. 3B). KEGG analysis states that supernumerary teeth were related to transcriptional misregulation in cancer (Fig. 3C). Interacting proteins for APC gene were CTNNB1, AXIN1, GSK3B, CSNK1E and ARHGEF4, which were very important factors in Wnt signalling pathway (Fig. 3D). We analysed expression levels of gene (CTNNB1, AXIN1, GSK3B, CSNK1E, ARHGEF4) from different supernumerary teeth patients. We found that when APC has mutation, CTNNB1 (β‐catenin) dependent classical Wnt signalling pathway anomaly, then result in abnormal tooth germ development, thereby forming Odontoma, Supernumerary teeth (Fig. S1).

### APC expression profile in human supernumerary teeth and ICR mouse tooth germ

**Figure 4:**
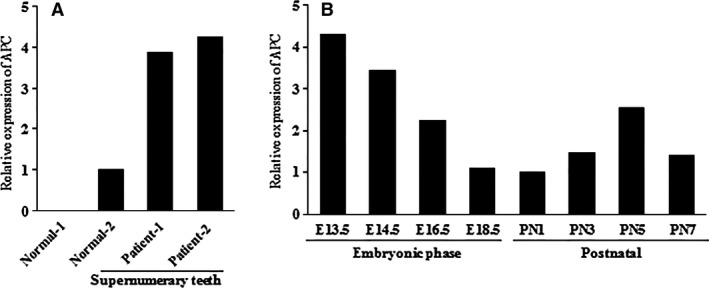
Adenomatous polyposis coli (APC) expression profile in human supernumerary teeth and ICR mouse tooth germ. (**A**) APC expression levels in human supernumerary teeth and normal teeth based on RNA‐seq; (**B**) APC expression profile analysis of mouse tooth germ development by RNA‐seq.

RNA‐seq analysis was performed to identify and visualize the expression of the APC gene. In human supernumerary teeth, we compared APC gene expression levels in patients with normal and supernumerary teeth. The results reveal significant differences in APC gene expression between human supernumerary teeth dental pulp stem cells and normal cells (Fig. 4A).

During the development of mouse tooth germ, we found that APC expression levels gradually declined at the embryonic phase (E13.5, E14.5, E16.5 and E18.5). At the postnatal phase, APC expression increased from PN1 to PN5, but the expression level at PN7 was reduced compared with PN5 (Fig. 4B). This result indicates that the APC gene plays an important role in the development of the mouse tooth germ, especially in the embryonic phase.

## Discussion

The Gardner syndrome family assessed in this study is a valuable resource for this disease and for further understanding the mechanism of supernumerary teeth and dental tumours. In this study, in addition to three characteristics of Gardner syndrome (intestinal polyps, multiple osteoma, and skin and soft tissue tumours), the proband and one uncle exhibited tumours, along with a plurality of permanently impacted teeth. The proband also had extra teeth and jaw osteoma. According to the history collected from the proband's father, mandibular abnormalities were also noted. RNA‐seq function pathway analysis state that supernumerary teeth were related to transcriptional misregulation in cancer. Dental anomalies, such as extra teeth, impacted teeth, congenital missing teeth, cementum hyperplasia, dental cysts and odontogenic tumours, are noted in 70% of Gardner syndrome individuals.

Till now, there are many clinical reports about supernumerary teeth 27, 28, 29, 30. But the disease pathogenesis is still unclear. Supernumerary teeth have complex genetic background and associated with many syndrome 31, 32.

In this study, the proband and his two uncles had multiple dental tumours. In addition, a shadow was noted around the film, mainly located in the anterior mandible. Although the World Health Organization still classifies a benign tumour of the tooth as an odontogenic tumour, these lesions are actually tooth tumour tissue malformations, not true tumours. Odontogenic tumour tissue from the tooth is present in different proportions and at different levels during the development of composition 33. Odontoma is generally divided into two types: combination and mixed. Numerous small tooth tumours can be aligned in an orderly manner 34. Mixed odontoma assumes no homogenous tooth shape and involves a group of toothless derived tissue‐specific structures 35. Pippi performed numerous studies on teeth. Many teeth tumours were retrospectively analysed; the author found that pointed teeth and teeth tumours have the same origin 36. Some scholars note that odontoma is directly attributable to the increase in teeth, the formation of teeth into cone‐shaped nodules, normal shape and tooth tumours 37. The results of this study also showed that APC mutations only cause tooth tumours and occasionally results in more teeth.

In our study, we identified an APC gene deletion (4292‐4293‐Del GA) in the proband's third uncle (II‐4), the proband (III‐1) and the proband's second uncle (II‐3). This mutation was not identified among the more than 1,000 types of mutations in the human APC gene database; thus, this mutation is a novel causative mutation of the APC gene.

The APC gene is located on chromosome 5q21‐q22 and contains 15 exons encoding a 2843‐amino acid APC protein 38. The APC protein is an important component of the Wnt signalling pathway. APC inhibits the Wnt signalling pathway in the gut, skin, immune system, bone tissue and brain tissue. In addition, APC plays an important role in the regulation of cell proliferation and differentiation by down‐regulating β‐catenin protein and preventing its nuclear translocation. In the context of APC loss of function, β‐catenin accumulates in the cytoplasm, and excessive β‐catenin is transferred to the nucleus to activate transcription factor family TCF/LEF binding members involved in cell proliferation and apoptosis 39. APC inhibits β‐catenin/TCF transcription through a variety of mechanisms. APC GSK3β and Axin form complexes to promote sequential phosphorylation, ubiquitination and eventual degradation of β‐catenin. APC facilitates the nuclear transport of β‐catenin and reduces β‐catenin/TCF levels in the nucleus. APC and β‐catenin binding prevents the interaction between β‐catenin and TCF. APC binding to βTrCP, CtBP, TLE1 and HDAC1 suppresses the formation of a stable complex, thus promoting CtBP‐mediated inhibition of the Wnt signalling pathway 40, 41, 42, 43.

APC gene plays an important role in the development of the tooth germ. Jiang indicates that during the bud stage, strong positive expression of APC protein was found in the oral epithelium and the dental lamina, but the expression displayed a down‐regulation tendency. The result of β‐catenin suggests its contribution in the early development of enamel organ and the proliferation of cell 44. Jae‐Young Kim found that APCDD1 modulates the gene expression of Wnt‐ and EK‐related signalling molecules at the cap stage of tooth development and is involved in tooth cusp patterning by modulating the epithelial rearrangement 45. Supernumerary tooth formation in mouse molar transplants 46. During the development of mouse tooth germ, we found that APC expression levels gradually declined at the embryonic phase (E13.5, E14.5, E16.5 and E18.5). At the postnatal phase, APC expression increased from PN1 to PN5, but the expression level at PN7 was reduced compared with PN5. Our result indicates that the APC gene plays an important role in the development of the mouse tooth germ, especially in the embryonic phase.

**Figure 5:**
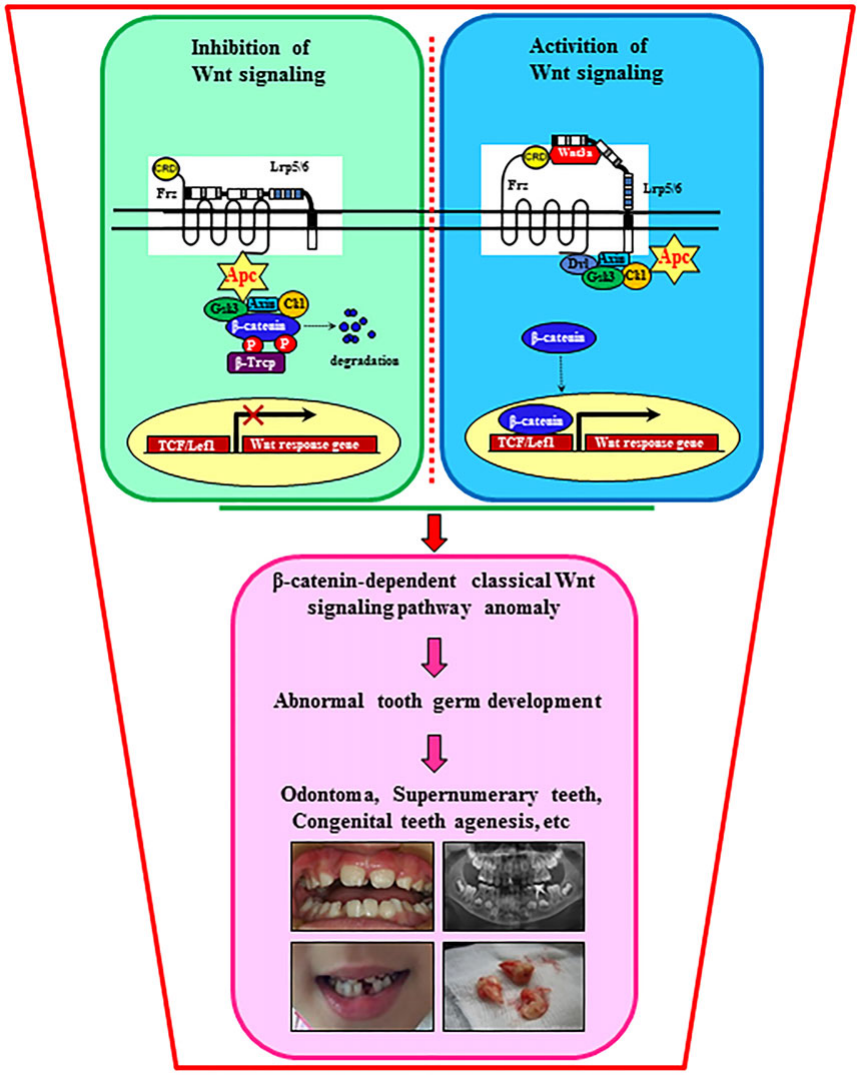
Hypothesis for supernumerary teeth pathogenesis.

We make a hypothesis for supernumerary teeth pathogenesis with Wnt: β‐catenin‐dependent classical Wnt signalling pathway anomaly, APC mutation result in abnormal tooth germ development, thereby forming Odontoma, Supernumerary teeth, Congenital teeth agenesis, *et al*. (Fig. 5).

Gardner syndrome treatment should primarily focus on intestinal polyps, which, if left untreated, will become cancerous 47. Gardner syndrome patients typically die due to intestinal polyps, and thus, surgery should be performed as soon as possible. Therefore, early diagnosis of Gardner syndrome is particularly important. Seehra and others recommended that FAP patients and their families undergo yearly sigmoidoscopy at 10–12 years of age 48. Given the presence of abnormal teeth and intestinal polyps, a dentist should be involved in the early diagnosis to prevent malignant intestinal polyps in Gardner syndrome 49. It is important to note that the part of the jaw inside the osteoma will continue to grow, and X‐ray examination on a regular basis is necessary for these patients 50. Gardner syndrome involves abnormal teeth, and doctors must develop a comprehensive oral treatment programme by coordinated operations 51.

Through an extensive literature search, we only found a limited number of foreign study on the jaw and teeth similar to the syndrome found in this family. In this study, an APC gene deletion was identified in the Gardner syndrome pedigree. The proband experienced a timely diagnosis of Gardner syndrome and early removal of intestinal polyps. This study has laid a solid foundation for our future in‐depth studies on teeth, dental tumours and the APC gene. We also shed light on the APC mechanism during tooth development.

Supernumerary teeth can be an important component of a distinctive disorder, and they can also be an important clue for early diagnosis. The early detection of abnormalities allows us to offer correct patient management, and this information is also important for making well‐informed decisions about long‐term medical care and treatment. In application, Makino investigates an *in vitro* immunoregulatory property of supernumerary tooth‐derived stem cells (SNTSCs) for T cells and shows an *in vivo* immune effect of SNTSCs in human systemic lupus erythematosus (SLE) model MRL/*lpr* mice 52.

We will combine tooth development and dental stem cells as well as animal models for further study *in vitro* and *in vivo*. Supernumerary tooth stem cells will play important part in cell‐based therapy for treatment of immune diseases and tooth regeneration. We will also use retrovirally packaged siRNA or overexpression to study the APC gene and other genes as well as signalling pathways involved in the formation of extra teeth.

## Conflict of interest

The authors declare that they have no competing interests and source of funding.

## Supporting information


**Figure S1** The expression levels of interacting proteins for APC in human supernumerary teeth and normal teeth.
