# Supplementary figures and images for: A novel mutation of adenomatous polyposis coli (APC) gene results in the formation of supernumerary teeth
Source: J Cell Mol Med. 2017 Aug 7;22(1):152–62. doi: 10.1111/jcmm.13303 (PMC5742724; doi:10.1111/jcmm.13303)

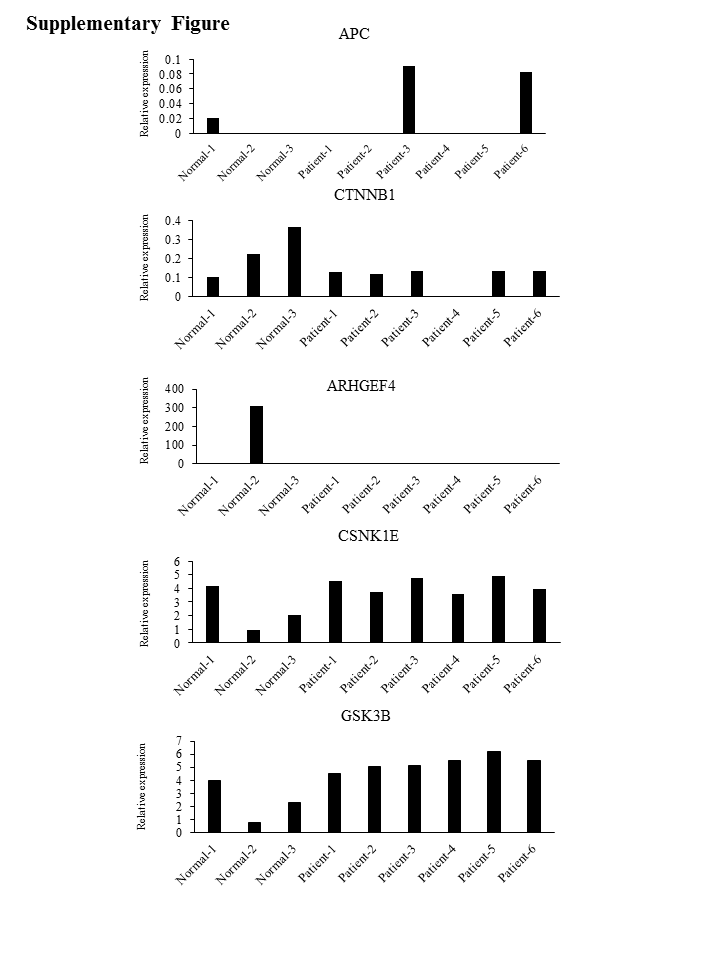

Supplement: Supplementary file 1 — Figure S1 The expression levels of interacting proteins for APC in human supernumerary teeth and normal teeth. [file JCMM-22-152-s001.TIF]
